# Diagnostic value of combined detection of cryptococcal antigen in serum and lung puncture fluid using lateral flow assay for diagnosing pulmonary cryptococcosis: a single-center prospective study

**DOI:** 10.3389/fmicb.2025.1747123

**Published:** 2026-01-16

**Authors:** Jinbao Huang, Xinchen Lin, Meiqin Jiang, Yangyu Li, Li Wang, Shungui Xu, Heng Weng, Hongyan Li, Ling Ye

**Affiliations:** 1 Department of Pulmonary and Critical Care Medicine, The Affiliated People’s Hospital of Fujian University of Traditional Chinese Medicine, Fuzhou, China; 2 Department of Interventional Radiology, The Affiliated People’s Hospital of Fujian University of Traditional Chinese Medicine, Fuzhou, China; 3 Department of Clinical Laboratory Medicine, The Affiliated People’s Hospital of Fujian University of Traditional Chinese Medicine, Fuzhou, China; 4 Department of Pathology, The Affiliated People’s Hospital of Fujian University of Traditional Chinese Medicine, Fuzhou, China; 5 Department of Critical Care Medicine, The Affiliated People’s Hospital of Fujian University of Traditional Chinese Medicine, Fuzhou, China

**Keywords:** cryptococcal antigen, diagnosis, immunochromatography, lateral flow immunoassay, pulmonary cryptococcosis

## Abstract

**Objective:**

To evaluate the diagnostic value of the combined detection of cryptococcal antigen (CrAg) in the serum and lung puncture fluid (LPF) using a lateral flow assay (LFA) in patients with pulmonary cryptococcosis (PC).

**Methods:**

Patients with suspected PC were prospectively enrolled between June 2023 and July 2025 and underwent the IMMY CrAg LFA (Immuno-Mycologics, Norman, OK, United States) of serum and LPF specimens. Conventional fungal cultures were performed simultaneously and the diagnostic efficiencies of these methods for PC were compared.

**Results:**

Samples were obtained from 167 patients, 33 with PC and 134 without PC. Among patients with PC, the sensitivity of serum CrAg testing was 75.8% [95% confidence interval (CI), 61.1–90.4%; 25/33] and that of LPF CrAg testing was 90.9% (95% CI, 81.1–100%; 30/33). The combination of serum and LPF testing increased the overall sensitivity of CrAg testing to 97.0% (95% CI, 91.2–100%; 32/33). The sensitivity, accuracy, and negative predictive value (NPV) of LPF culture were significantly lower than those of serum CrAg detection (*P* < 0.001, *P* = 0.002, and *P* < 0.001, respectively) and LPF CrAg detection (*P* < 0.001, *P* < 0.001, and *P* < 0.001, respectively); however, the diagnostic accuracy of serum and LPF CrAg detection did not differ significantly (*P* > 0.05). The sensitivity and NPV of serum and LPF CrAg detection combined were significantly higher than those of serum CrAg detection alone (*P* = 0.027 and *P* = 0.037, respectively). However, the accuracy of the two methods did not differ significantly (*P* = 0.061). Moreover, the area under the receiver operating characteristic curve for serum and LPF testing combined (0.981) was better than those for LPF culture (0.591), serum CrAg alone (0.875), and LPF CrAg alone (0.955).

**Conclusion:**

Serum CrAg testing has superior sensitivity and accuracy compared to the conventional cryptococcal culture method using LPF, and CrAg testing of LPF samples further enhances diagnostic sensitivity. Moreover, detecting CrAg in serum and LPF specimens together provides high sensitivity and specificity, which significantly improves the diagnostic efficiency of PC.

## Introduction

Pulmonary cryptococcosis (PC) is an invasive fungal infection of the lungs that results from the inhalation of *Cryptococcus neoformans* (*C. neoformans*) spores or yeast cells from the external environment (Botts and Hull, 2010). Cryptococcal infection has historically been considered a common acquired immune deficiency syndrome (AIDS)-defining opportunistic infection (May et al., 2016). However, recent evidence suggests a growing incidence among immunocompromised individuals without human immunodeficiency virus (HIV) infection (Coussement et al., 2023). In recent years, the incidence of PC in the Chinese population has steadily increased, and it is currently ranked third among pulmonary fungal diseases (Liu et al., 2011). Notably, the proportion of patients with apparently “normal immune function” ranges from 67.5 to 70.2% (Chen et al., 2021; Lan et al., 2016), which is considerably higher than the rates reported in other countries (21.0–35.2%) (Pappas et al., 2001; Coussement et al., 2023).

Differentiating PC from common lung diseases remains challenging because of its complex and heterogeneous clinical manifestations and radiological features. This often leads to delays in early diagnosis and increases the risk of misdiagnosis and inappropriate treatment (Lan et al., 2016), which may result in severe clinical consequences (Park et al., 2009; Setianingrum et al., 2019). On October 25, 2022, the World Health Organization designated *C. neoformans* as one of four priority fungal pathogens (World Health Organization, 2022), highlighting the need for strengthening preparedness and response to cryptococcal infections.

Due to inherent limitations in culture conditions and technical methodologies, conventional cryptococcal staining smears and culture techniques have low sensitivity and long turnaround times (TATs), thereby failing to adequately meet the diagnostic requirements of clinical practice (Shirley and Baddley, 2009). Historically, the diagnosis of PC has depended primarily on lung tissue biopsy and histopathological evaluation; however, this approach is associated with diagnostic delays and imposes high technical demands on the pathology staff (Zheng et al., 2021; Wang et al., 2022). Although lung biopsy has a relatively high success rate for acquiring pathological specimens from pulmonary lesions, false-negative results remain a concern. Notably, the nodular and mass-like features commonly observed in PC closely resemble those of malignant neoplasms. Consequently, in clinical practice, surgery is often required for definitive diagnosis and management (Setianingrum et al., 2019). Therefore, novel, rapid, accurate, and minimally invasive diagnostic methods are urgently required.

*In vitro* studies have demonstrated that the capsule is a critical virulence factor for *C. neoformans*, with capsular polysaccharides (CPs) capable of dissociating from the cell surface and dispersing into the surrounding growth environment (May et al., 2016). Detection of *Cryptococcus* CP antigens in clinical specimens is a valuable tool for diagnosing cryptococcal infections. Recently, tests that detect serum cryptococcal antigen (CrAg) have substantially improved the diagnostic efficiency of PC. Lateral flow assay (LFA) has been widely adopted for CrAg detection in clinical settings because of its user-friendly operation, high specificity (97.7–100%) (Yan et al., 2021; Zeng et al., 2021; Hu et al., 2022), and high sensitivity (75.0–85.1%) (Liu et al., 2011; Zeng et al., 2021; Hu et al., 2022; Shi et al., 2023). Nevertheless, although the serum CrAg test plays an important diagnostic role, a considerable number of false-negative results persist, particularly among patients without HIV infection in whom the false-negative rate may exceed 30% (Yan et al., 2021). A multicenter prospective study conducted in China (Chen et al., 2021) demonstrated that the sensitivity of serum CrAg detection in patients without HIV infection with isolated PC was 70.7%, which was significantly lower than that observed in patients with PC and extrapulmonary disseminated infections, primarily cryptococcal meningitis (CM) (100%). The serum CrAg positivity rate was lower (approximately 60%) in individuals with solitary pulmonary lesions or asymptomatic infections. Therefore, serum CrAg testing has limitations in diagnosing PC, particularly in individuals without HIV infection, organ transplantation, or other forms of severe immunodeficiency and in those with non-disseminated disease. Clinicians should be aware of the potential for false negative results.

The sensitivity and specificity of CrAg testing in the cerebrospinal fluid (CSF) of patients with CM are significantly higher than those in serum (Xie et al., 2017). In patients with PC with small pulmonary nodules (≤2.5 cm), the CrAg positivity rate in bronchoalveolar lavage fluid (BALF) exceeds that in serum (Oshima et al., 2018). These findings suggest that CrAg concentration and detection sensitivity increase in proximity to the site of infection. As most PC lesions are located in the peripheral regions of the lungs, percutaneous lung biopsy (PLB) has become a commonly used and effective diagnostic approach (Pappas et al., 2001; Lan et al., 2016). Lung puncture fluid (LPF) is directly aspirated from the lesion site, and CrAg may be more readily detected in LPF during the early stages of the disease, particularly when the lesion is small. The integration of CrAg detection technology with PLB may serve as a valuable adjunct for early PC diagnosis. Therefore, this study aimed to evaluate the diagnostic utility of combined CrAg testing of serum and LPF samples from HIV-negative patients with PC who had not undergone bone marrow or organ transplantation, using IMMY CrAg LFA (Immuno-Mycologics, Norman, OK, United States).

## Materials and methods

### Patients

Patients who underwent CrAg testing between June 2023 and July 2025 at the Affiliated People’s Hospital of Fujian University of Traditional Chinese Medicine were prospectively enrolled. The inclusion criteria were as follows: (1) age ≥ 18 years; (2) a suspected diagnosis of PC; and (3) serum and LPF specimens had undergone CrAg testing. The exclusion criteria were: (1) patients with contraindications for PLB, such as severe cardiopulmonary insufficiency, impaired coagulation function, or active massive hemoptysis, and (2) patients with HIV infection or a history of bone marrow or organ transplantation. Patients with missing data on key variables or unclear diagnoses were excluded from the analysis.

This study was conducted in accordance with the Declaration of Helsinki and was approved by the Ethics Committee of the Affiliated People’s Hospital of Fujian University of Chinese Medicine (Approval No. 2023-042-02).

### Diagnostic criteria for PC and non-PC

The standardized diagnostic criteria for PC are as follows (Weng, 2010; Zhou et al., 2017; Donnelly et al., 2020; Chang et al., 2024): (1) necessary conditions, defined as detection of *Cryptococcus* by pathological examination of lung tissue biopsy or smear/culture of lung puncture specimens (tissue, LPF) and (2) secondary conditions, defined as presence of typical clinical features and imaging findings of PC and receiving effective anti-cryptococcal therapy. The clinicians were completely blinded to the serum and LPF CrAg test results during diagnosis. The final diagnosis of PC was based on a comprehensive evaluation of all available medical data, excluding the knowledge of CrAg test results. A confirmed diagnosis must satisfy the necessary criteria.

For the non-PC group, the diagnosis (such as lung cancer or pneumonia) was confirmed through a comprehensive clinical evaluation incorporating symptoms, physical examinations, various laboratory tests, imaging manifestations, cytological and pathological findings, and evidence of effective drug treatments (Liu et al., 2024; Huang et al., 2025).

### Specimen collection for CrAg detection

(1) LPF samples: PLB was performed under chest computed tomography (CT) guidance in accordance with established procedural standards. Two to four tissue samples were obtained from the pulmonary lesions. Prior to the procedure, a 5 mL sterile test tube pre-filled with sterile normal saline was prepared. Following each biopsy, the tissue specimens were immediately fixed in a 4% formaldehyde solution. The cutting needle, including its tip containing the residual tissue and puncture fluid, was then immersed in a saline-containing test tube and gently agitated to ensure thorough dispersion of the residual material. After centrifugation at 3,000 × *g* for 10 min, the supernatant was collected for subsequent testing. (2) Serum samples: Prior to antifungal therapy, approximately 3 mL of fasting venous blood was collected from the antecubital vein on the same day as the PLB and transferred to a sterile non-coagulant tube. The sample was centrifuged at 3,000 × *g* for 10 min, and the resulting serum was separated for subsequent laboratory analysis.

### *Cryptococcus* smear and culture and other pathogen detection

Sterile human specimens, including LPF, blood, CSF, pleural effusion, and lung puncture tissue (with specimen grinding), were collected and injected into blood culture bottles, which were then incubated and monitored using the BACTEC FX Automated Blood Culture System (Becton, Dickinson and Company, United States). To detect positive signals, the culture bottles were promptly subcultured on blood agar plates. Direct smears were prepared and stained with either Gram stain or India ink for preliminary microscopic examination. Based on the smear findings, specimens were inoculated onto Sabouraud dextrose agar plates for fungal isolation. Following colony growth, identification was performed using a CombiPlate for colorimetric differentiation. Additionally, a bacterial suspension adjusted to a turbidity of 1.8–2.0 McFarland units was prepared for analysis by matrix-assisted laser desorption/ionization time-of-flight mass spectrometry (VITEK MS, bioMérieux, France) or using the VITEK YST fungal identification card (bioMérieux, France). Additional pathogen testing was also performed on the LPF. Sputum and BALF samples were screened for *Cryptococcu*s infection.

### Serum and LPF CrAg testing by LFA

**FIGURE 1:**
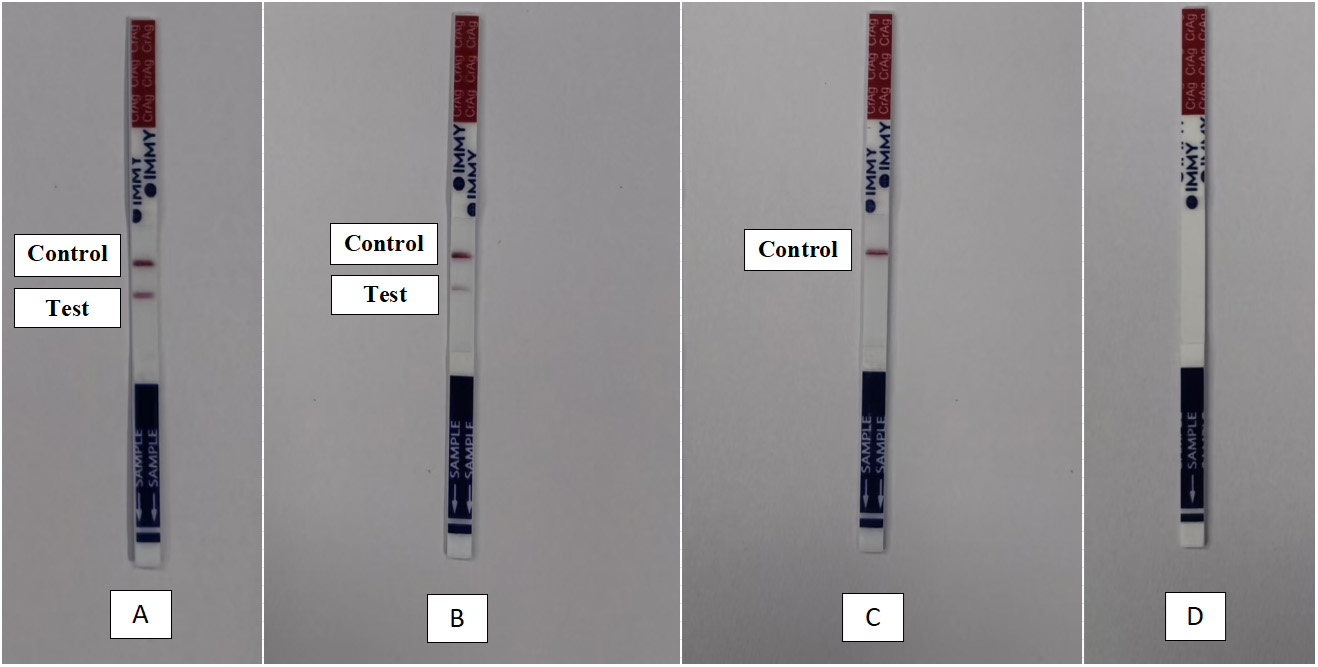
Schematic diagram of cryptococcal antigen test results obtained by lateral flow assay. **(A)** Positive, **(B)** weakly positive, **(C)** negative, and **(D)** invalid.

The United States Food and Drug Administration (US FDA) has approved the use of IMMY CrAg LFA for diagnosing cryptococcal infections (Liu et al., 2022; Chang et al., 2024). In this study, serum was collected from whole blood samples, and the supernatant was obtained from LPF from all patients under the principle of blinding. All serum and lung aspirate specimens were tested for the presence of CrAg using the IMMY CrAg LFA, according to the manufacturer’s instructions. The testing procedure was as follows: one drop of sample diluent was added to a sterile test tube. Next, 40 μL of serum or the supernatant from LPF was transferred into the test tube and mixed thoroughly with the diluent. The white end of the cryptococcal antigen test strip was immersed in the prepared sample solution. The test was allowed to develop for 10 min before interpreting the results. A positive outcome was indicated by the appearance of two distinct bands (test and control) (Figures 1A,B), whereas a negative result was indicated by the presence of the control band only (Figure 1C). If a control band failed to appear, the test was considered invalid and repeated (Figure 1D).

### Statistical analysis

IBM SPSS Statistics (version 19.0; IBM Corp., Armonk, NY, United States) was used for statistical analysis. Continuous and categorical data are expressed as medians (interquartile ranges) and percentages (%), respectively. The performance of each testing method was expressed in terms of sensitivity, specificity, accuracy, positive predictive value (PPV), and negative predictive value (NPV). The diagnostic performance of the different methods was compared using the chi-squared test or Fisher’s exact test. Receiver operating characteristic (ROC) curve analysis was used to compare the diagnostic efficiencies of the different methods based on the area under the curve (AUC). For the combined serum and LPF CrAg testing, a positive result was defined as positivity in either the serum or LPF CrAg test. The diagnostic performance of this composite binary rule was evaluated, and no *post hoc* threshold optimization was applied. Statistical significance was set at *P* < 0.05.

## Results

### Patient characteristics

**FIGURE 2:**
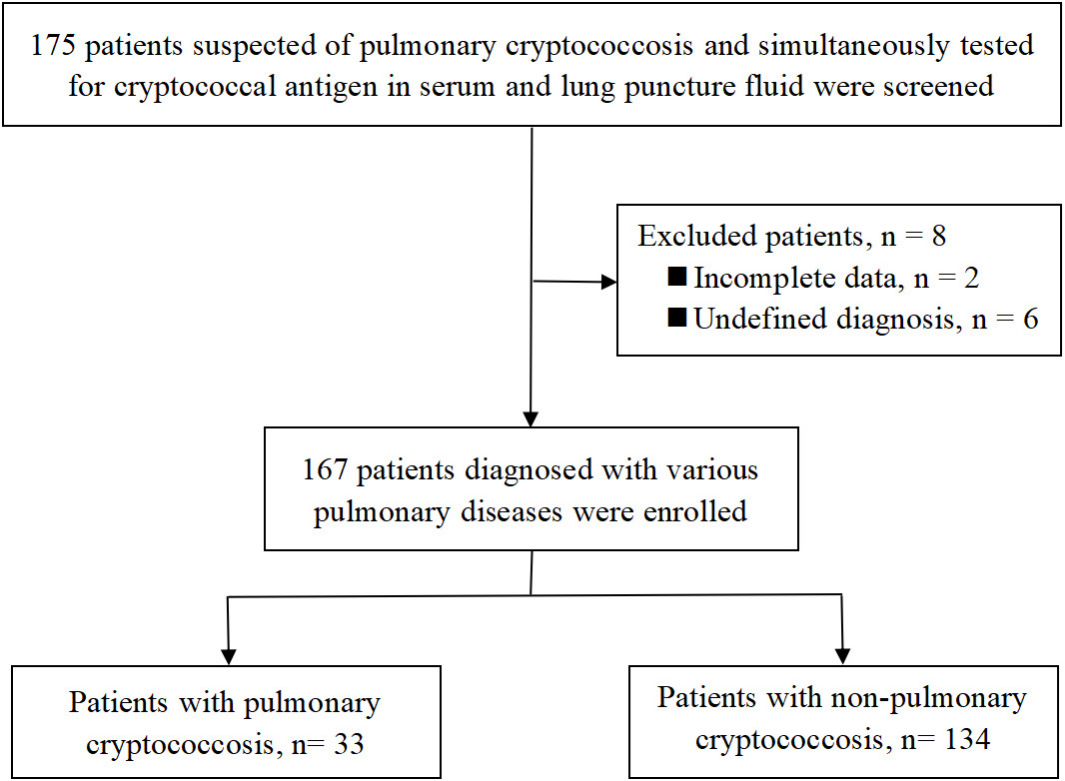
Flowchart of the enrolled patients.

Serum and LPF samples from 175 patients with suspected PC were simultaneously tested using CrAg LFA (Figure 2). After excluding 8 patients who did not meet the eligibility criteria, 167 patients were included in the analysis, including 33 with PC and 134 without PC.

All patients with PC were diagnosed by histopathological examination of the lung tissue and/or culture of *Cryptococcu*s from the LPF/lung tissue specimens. Three patients had PC combined with cryptococcal infections at other sites, and the remaining 30 had isolated PC. The clinical characteristics of the patients are presented in Table 1, and the detection results for *Cryptococcus* in the different specimens are presented in Table 2.

**TABLE 1:** Clinical characteristics of patients with pulmonary cryptococcosis (*N* = 33).

Clinical characteristics	Value
Age, mean (range), years	53 (45–63)
Sex, male, n (%)	18 (55)
Underlying disease, n (%)	22 (67)
Immunosuppressive disease, n (%)	17 (52)
Malignant lymphoma	4 (12)
Lung cancer with chemotherapy and immunotherapy	1 (3)
Chronic lymphocytic leukemia	1 (3)
SLE treated with glucocorticoid and immunosuppressant therapy	2 (6)
COPD treated with long-term systemic glucocorticoid therapy	1 (3)
Diabetes mellitus	8 (24)
Isolated diabetes	6 (75)
Concomitant myelodysplastic syndrome with ring sideroblasts and secondary myelofibrosis	1 (13)
Concomitant pemphigus erythematosus treated with systemic glucocorticoid therapy	1 (13)
**Symptoms, n (%)**	
Cough	9 (27)
Expectoration	6 (18)
Hemoptysis	1 (3)
Fever	1 (3)
Headache	1 (3)
Asymptomatic	23 (70)
**Laboratory results, n (%)**	
White blood cell count, > 9.5 × 10^9^/L	2 (6)
White blood cell count, < 3.5 × 10^9^/L	0 (0)
Leukomonocyte count, < 1.1 × 10^9^/L	7 (21)
C-reactive protein, > 10 mg/L	7 (21)
Positive rate of 1, 3-β-D-glucan antigen test	0 (0)
Positive rate of galactomannan antigen test	0 (0)
Non-antifungal therapy before diagnosis, n (%)	6 (18)
Antibacterial therapy	5 (83)
Antituberculous therapy	1 (17)

COPD, chronic obstructive pulmonary disease; SLE, systemic lupus erythematosus. Data are presented as n (%) or mean (range).

**TABLE 2:** *Cryptococcu*s detection results in patients with pulmonary cryptococcosis.

Detection means	Number of specimens	Positive result (n, %)
Pathological examination of lung tissue	33	31 (94)^a^
Serum CrAg	33	25 (76)
LPF CrAg	33	30 (91)
BALF CrAg	3	1 (33)
Cerebrospinal fluid CrAg	3	3 (100)
LPF culture	33	6 (18)
Lung tissue culture	5	1 (20)
BALF culture	5	1 (20)
Sputum culture	9	1 (11)
Cerebrospinal fluid culture	3	3 (100)^b^
Hydrothorax culture	1	1 (100)
Culture of all lung specimens	52	9 (17)
Culture of all human specimens	56	13 (18)
Lung tissue mNGS	3	2 (67)
BALF tNGS	1	0 (0)

BALF, bronchoalveolar lavage fluid; CrAg, cryptococcal antigen; LPF, lung puncture fluid; mNGS, metagenomic next-generation sequencing; tNGS, targeted next-generation sequencing. Data are presented as n (%). ^a^Five patients had inconclusive initial pathological findings regarding cryptococcal infections. Following consultation with pathologists at a higher-level hospital, these cases were confirmed as PC. Pathological results were inconclusive in two additional cases; however, *Cryptococcus* was isolated from the LPF cultures. ^b^Refers to cerebrospinal fluid specimens collected from patients with confirmed cryptococcal meningitis.

The non-PC group comprised patients with primary lung malignancies (69 cases, 51.5%), lung metastases (15 cases, 11.2%), bacterial pneumonia (16 cases, 11.9%), pulmonary tuberculosis (PT) (11 cases, 8.2%), lung abscess (5 cases, 3.7%), silicosis (5 cases, 3.7%), organizing pneumonia (3 cases, 2.2%), pulmonary aspergillosis (3 cases, 2.2%), and benign lung tumors (3 cases, 2.2%). The median age was 67 years (interquartile range: 58–72 years), and of the 134 patients, 79 (59.0%) were male.

### Results of CrAg testing

**FIGURE 3:**
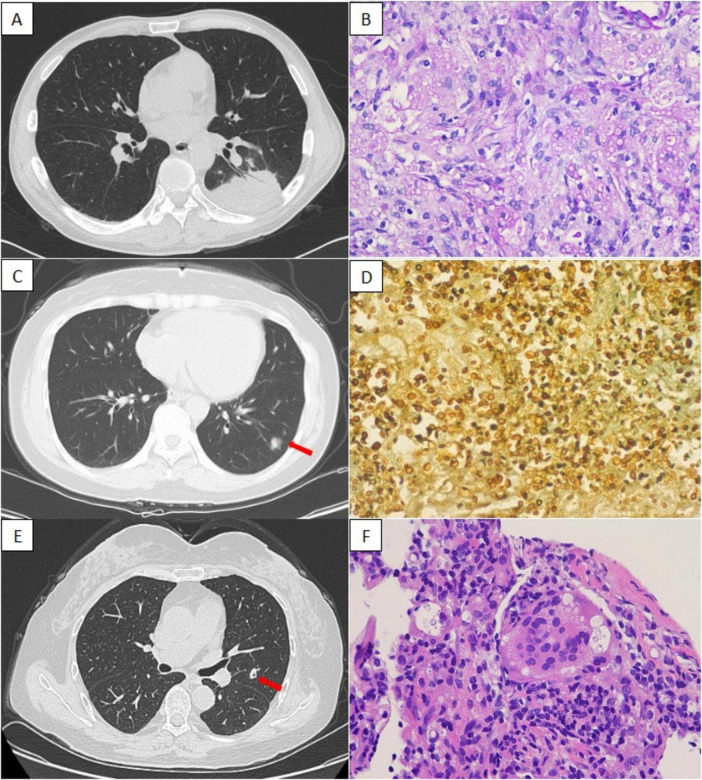
Patients with typical pulmonary cryptococcosis with negative serum CrAg test results. *Case 1*: A 49-year-old man with extensive consolidation in the left lower lobe on chest CT **(A)**. Both serum and bronchoalveolar lavage fluid CrAg tests were negative. Subsequently, a PLB confirmed cryptococcal infection **(B)**, and the LPF tested positive for CrAg. *Case 2*: A 35-year-old woman with multiple nodular shadows in the left lower lobe on chest CT (**C**, arrow). The PLB confirmed cryptococcal infection **(D)**, and the LPF CrAg test result was positive. *Case 3*: A 67-year-old woman with a solitary cavitary nodule in the left lower lobe on chest CT (**E**, arrow). Histopathological examination of a PLB specimen confirmed cryptococcal infection **(F)**, and the LPF CrAg test result was positive. *Cryptococcu*s species were isolated from the LPF culture. CrAg, cryptococcal antigen; CT, computed tomography; LPF, lung puncture fluid; PLB, percutaneous lung biopsy.

**FIGURE 4:**
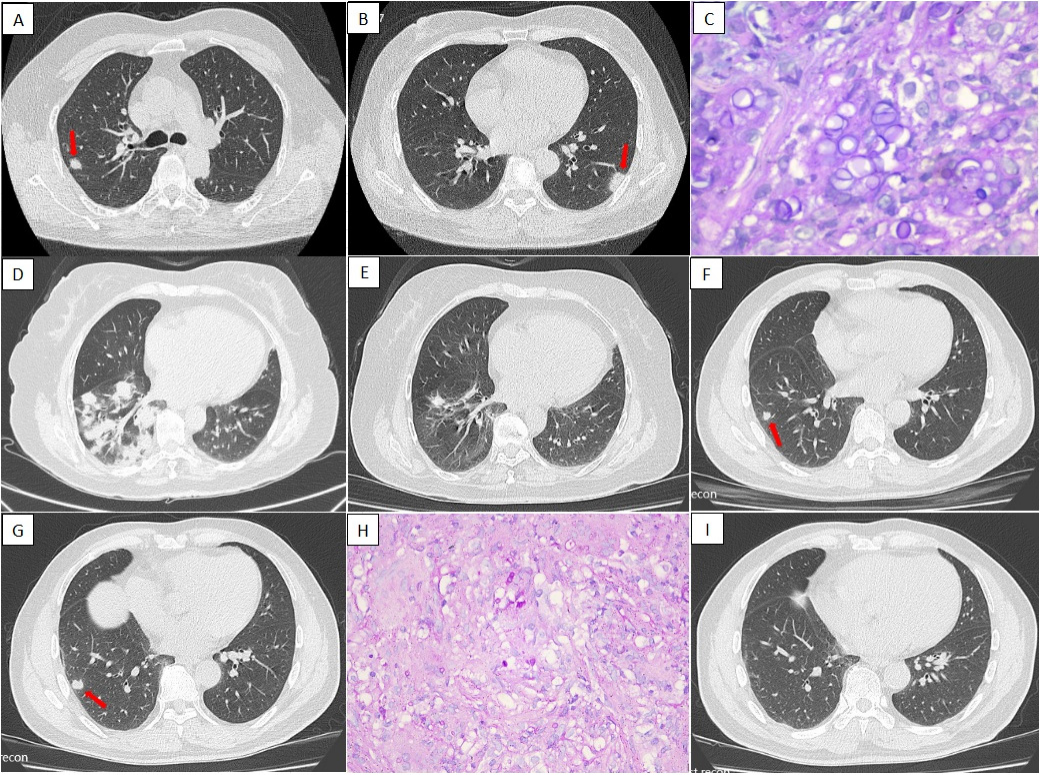
Three patients with pulmonary cryptococcosis with negative LPF CrAg test results. *Case 1*: A 53-year-old woman with multiple pulmonary nodules on chest CT (**A,B**, arrows). Serum CrAg testing was positive, and histopathological examination of a PLB specimen confirmed cryptococcal infection **(C)**. *Case 2*: A 66-year-old woman with multiple nodules and patchy consolidation in the right lung on chest CT **(D)**. Serum CrAg testing was positive and histopathology of a PLB specimen revealed *Cryptococcus*. Following 9 months of fluconazole therapy, the lesions had largely resorbed **(E)**. *Case 3*: A 63-year-old man with multiple nodules in the right lower lobe on chest CT (**F,G**, arrows). Serum CrAg testing was negative; however, histopathological analysis of a PLB sample revealed cryptococcal infection **(H)**. After antifungal therapy, the nodules resolved **(I)**. LPF, lung puncture fluid; CrAg, cryptococcal antigen; CT, computed tomography; PLB, percutaneous lung biopsy.

Of the 33 patients with PC, serum CrAg results were positive in 25 patients (75.8%) (Supplementary Figure 1), and 30 patients (90.9%) tested positive on parallel LPF CrAg testing (Tables 2, 3). Of the 8 patients with negative serum CrAg results, 5 had positive LPF CrAg results (Figure 3), and two of the 3 patients with negative LPF CrAg results had positive serum CrAg results (Figures 4A–F). All but one patient with PC had positive serum and/or LPF CrAg results, and one patient had negative results for both serum and LPF CrAg tests (Figures 4G–I). Of the 3 patients whose BALF samples were submitted for CrAg testing, only one had a positive result. Of the 3 patients with PC and CM, the CSF was positive on CrAg testing in all 3 patients (Supplementary Figure 2). Among the 134 patients without PC, all LPF CrAg results were negative, and only one patient with PT exhibited a weakly positive serum CrAg result (Figure 1B). This patient had negative LPF CrAg, negative cultures, and negative follow-up serum CrAg results after anti-tuberculosis treatment, and responded to tuberculosis therapy without antifungal treatment. This comprehensive clinical follow-up confirmed that the initial result was a false positive.

**TABLE 3:** Comparison of different methods for the diagnosis of pulmonary cryptococcosis.

Testing methods	PC (*N* = 33)		Without PC (*N* = 134)		Sensitivity (%)	Specificity (%)	Accuracy (%)	PPV (%)	NPV (%)
	Positive	Negative	Positive	Negative					
LPF culture	6	27	0	134	18.2 (5.1, 31.3)*	100 (97.3, 100)	83.8 (78.3, 89.4)	100 (54.1, 100)	83.2 (77.5, 89.0)
Serum CrAg	25	8	1	133	75.8 (61.1, 90.4)	99.3 (96.2, 100)	94.6 (91.2, 98.1)	96.2 (81.6, 99.9)	94.3 (90.5, 98.1)
LPF CrAg	30	3	0	134	90.9 (81.1, 100)	100 (97.3, 100)	98.2 (96.2, 100)	100 (88.4, 100)	97.8 (95.4, 100)
Combined serum and LPF CrAg**	32	1	1	133	97.0 (91.2, 100)	99.3 (97.8, 100)	98.8 (97.1 100)	97.0 (91.2, 100)	99.3 (97.8, 100)

*95% confidence interval. Data are presented as n (%). Significance was determined using the chi-squared test or Fisher’s exact test. ^**^A positive result was defined as a positive CrAg test result detected in either serum or LPF. CrAg, cryptococcal antigen; LPF, lung puncture fluid; NPV, negative predictive value; PC, pulmonary cryptococcosis; PPV, positive predictive value.

A comparison of patients with PC with positive and negative serum CrAg test results revealed no statistically significant differences between the two groups in terms of immune status, clinical manifestations, or chest imaging features (Supplementary Table 1). Although the proportion of patients with solitary lesions was higher among those with negative serum CrAg results than those with positive results (50.0% vs. 24.0%, respectively), the difference was not statistically significant (*P* = 0.205). Similarly, among patients with PC presenting with nodular mass shadows, the incidence of solitary nodules was markedly higher in patients with negative serum CrAg results than those with positive results (60.0% vs. 15.8%); however, this difference was not statistically significant (*P* = 0.078).

### Comparison of different detection methods for diagnosing PC

The diagnostic performance of the four laboratory methods (LPF culture, serum CrAg testing, LPF CrAg testing, and serum and LPF CrAg testing combined) for PC is shown in Table 3. The sensitivity, accuracy, and NPV of LPF culture were significantly lower than those of serum CrAg testing (*P* < 0.001, *P* = 0.002, and *P* < 0.001, respectively) and LPF CrAg testing (*P* < 0.001, *P* < 0.001, and *P* < 0.001, respectively). However, the sensitivity, accuracy, and NPV of serum and LPF CrAg testing did not differ significantly. The sensitivity and NPV of the combined serum and LPF CrAg testing were significantly higher than those of serum CrAg testing alone (*P* = 0.027 and *P* = 0.037, respectively); however, the accuracy of the two methods did not differ significantly (*P* = 0.061). Moreover, no significant differences were observed in the sensitivity, accuracy, and NPV of LPF CrAg testing alone and combined serum and LPF CrAg testing. The specificity and PPV of the four detection methods did not differ significantly. Because approximately half of the non-PC patients had primary lung cancer, a subgroup analysis of specificity was performed. The specificities of serum and LPF CrAg tests in the non-primary lung cancer control subgroup were 98.5% (95% CI: 95.4–100%; 64/65) and 100% (95% CI: 95.5–100%; 65/65), respectively, comparable to those in the full control group at 99.3% (95% CI: 96.2–100%; 133/134) for serum CrAg and 100% (95% CI: 97.3–100%; 134/134) for LPF CrAg.

### ROC curves of different detection methods for diagnosing PC

**FIGURE 5:**
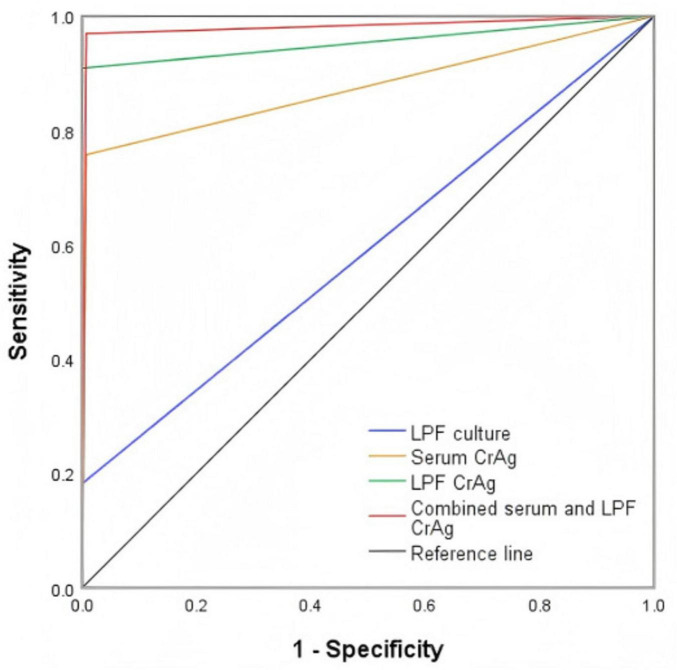
Receiver operating characteristic curve of the four testing methods for the diagnosis of PC. Compared with the standard diagnostic tests for PC, the sensitivity and specificity of LPF culture, serum CrAg, LPF CrAg, and combined serum and LPF CrAg were 18.2% (95% CI, 5.1–31.3%)/100% (95% CI, 97.3–100%), 75.8% (95% CI, 61.1–90.4%)/99.3% (95% CI, 96.2–100%), 90.9% (95% CI, 81.1–100%)/100% (95% CI, 97.3–100%), and 97.0% (95% CI, 91.2–100%)/99.3% (95% CI, 97.8–100%), respectively. CI, confidence interval; CrAg, cryptococcal antigen; LPF, lung puncture fluid; PC, pulmonary cryptococcosis.

As shown in Figure 5, the diagnostic values of LPF culture, serum CrAg testing, LPF CrAg testing, and combined serum and LPF CrAg testing for PC were compared using ROC curves. The AUCs were 0.591 (95% CI, 0.473–0.709; *P* = 0.106) for LPF culture, 0.875 (95% CI, 0.786–0.964; *P* < 0.001) for serum CrAg testing, 0.955 (95% CI, 0.897–1.000; *P* < 0.001) for LPF CrAg testing, and 0.981 (95% CI, 0.946–1.000; *P* < 0.001) for combined serum and LPF CrAg testing.

### Adverse reactions associated with PLB and prognostic outcomes

Among the 167 patients, 8 (4.8%) experienced mild hemoptysis, which resolved spontaneously or following hemostatic treatment. 10 patients (6.0%) developed pneumothorax, of which eight were classified as mild pneumothorax (<30% lung collapse) and resolved with oxygen therapy, and two patients had moderate pneumothorax (30–50% lung collapse), both of which resolved following closed thoracic drainage combined with oxygen inhalation. Of the 33 patients with PC, 32 experienced clinically significant improvement or achieved complete recovery following treatment, and one patient with CM and pleurisy in addition to PC died due to secondary severe aspiration pneumonia caused by *Klebsiella pneumoniae* (Supplementary Figures 2C,D).

## Discussion

Cryptococcal infections can affect various parts of the body, with a predilection for the central nervous system and the lungs (Chang et al., 2024). Previously, PC was considered a rare opportunistic infection; however, its incidence has increased. Among patients with AIDS, the annual incidence of cryptococcosis ranges from 80% to 90% (Shi, 2009). Furthermore, individuals without HIV infection are increasingly being recognized as a population at risk of developing cryptococcosis (May et al., 2016). According to studies, PC accounts for approximately 20% of all pulmonary fungal infections (Shi, 2009). The increased diagnosis of PC is primarily attributable to an increased number of individuals with immune deficiency, heightened awareness of PC among healthcare professionals, and advancements in diagnostic techniques (Chen et al., 2025). As demonstrated in the present study, among patients with PC without HIV infection, bone marrow transplantation, or organ transplantation, the proportion with normal immune function is similar to the proportion with immunosuppression. The clinical manifestations were non-specific, with imaging abnormalities observed in a high proportion of cases (69.7%). The chest CT findings exhibited diverse patterns, including nodules, masses, infiltrative consolidation, or a combination of multiple morphological lesions. Patients included in this cohort with suspected PC presented with various pulmonary conditions, such as lung malignancies, pneumonia, PT, and OP, which are clinically challenging to differentiate from PC. Prior to diagnosis, patients may have undergone various treatments, including antibiotics, antituberculous therapy, or treatment with glucocorticoids (Lan et al., 2016), which not only increases the risk of drug-related adverse reactions and imposes additional medical costs but also leads to severe consequences, such as extrapulmonary dissemination of the disease, due to delayed initiation of appropriate therapy. Furthermore, some patients undergo unnecessary surgery due to misdiagnosis of PC as lung tumors, resulting in physical harm and increased economic burden, as well as the potential risk of life-threatening intracranial fungal dissemination precipitated by surgery (Lai et al., 2005). Hence, early diagnosis of PC is critical.

Traditional diagnostic approaches for PC rely primarily on histopathological examinations in conjunction with special staining techniques. Su et al. (2022) reported that PLB had a higher diagnostic yield than transbronchial lung biopsy in patients with PC. In this study, the positive rate of PLB pathology was 94%, with TAT for pathological results ranging from 5 to 21 days. The initial pathological findings were inconclusive in 5 of the 33 patients. A definitive diagnosis of cryptococcal infection is achieved only after consultation with pathologists at a high-level medical institution, resulting in a considerable delay in diagnosis. Therefore, pathological examination not only entails a relatively prolonged diagnostic process but is also influenced by various factors, including the quality of biopsy specimens, staining quality of the pathology slides, variations in expertise and proficiency among staff making the diagnosis, and disparities in diagnostic capabilities between medical institutions, all of which may contribute to false-negative outcomes. Furthermore, this study demonstrated that the positive rates of *Cryptococcus* cultures in LPF, all lung specimens, and all human specimens were consistently below 20%, which is similar to the findings of Su et al. (2022). In a multicenter study conducted by Chen et al. (2021), the positivity rates for both *Cryptococcus* smears and cultures in respiratory specimens were even lower (<10%). Another multicenter retrospective study from the southeastern provinces of China also confirmed a low positivity rate of traditional fungal cultures (Shi, 2009). Compared to the relatively high detection rate of *Cryptococcus* in CSF (86.67%) (Chen et al., 2021), the sensitivity of detection in lung specimens is substantially lower, limiting their diagnostic utility.

The advent of CrAg detection methods has transformed the diagnostic landscape for cryptococcal infections. Currently, the three most widely used methods for CrAg detection in clinical practice are the latex agglutination test (LAT), enzyme-linked immunosorbent assay (ELISA), and LFA (Perfect and Bicanic, 2015; Su et al., 2022). Owing to their relatively complex procedures, prolonged processing times, stringent instrumentation requirements, and high operational costs, LAT and ELISA are not routinely used in most medical institutions. In contrast, LFA, a recently developed method for CrAg detection, has been increasingly adopted in clinical settings owing to its simplicity, rapidity, minimal infrastructure requirements, low cost, and strong concordance with both LAT (Perfect and Bicanic, 2015; Cáceres et al., 2017) and ELISA (Chinese Thoracic Society of Chinese Medical Association, 2017). CrAg testing is primarily performed on serum and CSF, and has also been performed using other clinical specimens, such as BALF, pleural effusion, and urine, with varying diagnostic performances (Oshima et al., 2018; Boyd et al., 2022; Chen et al., 2025). The diagnostic value of serum CrAg in PC is well-established. However, false negative results are common in the early stages of the disease (Perfect and Bicanic, 2015), in patients with small pulmonary lesions (Oshima et al., 2018), those with isolated lesions (Hu et al., 2022), non-immunosuppressed individuals (Su et al., 2022), and patients without extrapulmonary dissemination (Chen et al., 2021). Several studies have demonstrated that the sensitivity of CrAg testing in BALF is higher than that in serum specimens (Zeng et al., 2021), suggesting its potential as a supplementary specimen type for diagnosing PC (Chinese Thoracic Society of Chinese Medical Association, 2017). However, research on the detection of CrAg in BALF is limited (Chinese Thoracic Society of Chinese Medical Association, 2017), and test outcomes are substantially influenced by various factors, including lesion location, lesion size, selection of lavage site, and BALF volume. Therefore, the diagnostic value of CrAg testing of BALF for diagnosing PC remains unclear. As *C. neoformans* proliferates in the lungs of patients with PC, its CP is continuously secreted into the extracellular space and is subsequently disseminated into the bronchoalveolar and systemic circulation. Given the site of active fungal replication, it has been hypothesized that the concentration of CrAg is higher in the lung lesion tissue than in the blood or adjacent lung parenchyma. An exploratory prospective study of eight patients with PC (Liaw et al., 1995) demonstrated that when LAT was used to simultaneously detect CrAg in both serum and LPF, CrAg was detected in the LPF of all 8 patients but was detected in the serum of only three patients. Another recent two-center retrospective study (Hu et al., 2022) demonstrated that in a cohort of 47 patients with PC, the simultaneous detection of serum and LPF CrAg using IMMY CrAg LFA revealed that the sensitivity of LPF testing was higher than that of serum testing [98% (46/47) versus 77% (36/47)]. The specificity of both specimen types was 100%. These studies demonstrate that the diagnostic efficacy of LPF CrAg testing is greater than that of serum CrAg testing.

In this study, the sensitivity, accuracy, and NPV of serum and LPF CrAg tests for PC diagnosis were significantly higher than those of LPF cryptococcal cultures. Furthermore, the diagnostic sensitivity of LPF CrAg testing was higher than that of serum CrAg testing [90.9% (95% CI, 81.1–100%) vs. 75.8% (95% CI, 61.1–90.4%)], which is consistent with the results reported by Hu et al. (2022). Three of the 33 patients with PC had false-negative CrAg LFA results for LPF specimens, and the detection sensitivity of LPF in this study was slightly lower than that reported in previous studies (Liaw et al., 1995; Hu et al., 2022), which may be attributable to the higher dilution volume of the LPF (5 mL in this study compared with ≤ 1 mL in previous studies) and the possible inadequate volume of undiluted LPF obtained from some patients (Hu et al., 2022), potentially leading to a reduced antigen concentration. This study demonstrated that when both serum and LPF were tested for CrAg in parallel, the sensitivity of the combined test increased to 97.0% (95%CI, 91.2–100%), which was significantly higher than that achieved using serum samples alone. Among PC patients with negative serum CrAg results, five of eight tested positive in the LPF CrAg test, whereas among PC patients with negative LPF CrAg results, 2 of 3 had positive serum CrAg results. Testing the two sample types in parallel is complementary, and when used together, it substantially improves the diagnostic sensitivity. Previous studies have demonstrated that the proportion of patients with single lesions is higher among those with negative serum CrAg results than among those with positive serum CrAg results (Chen et al., 2021; Hu et al., 2022). In this study, the incidence of single lesions and isolated nodules in patients with negative serum CrAg test results was two- and three-fold higher, respectively, than that in patients with positive serum CrAg test results. Although false negative results on serum testing are more common during the early stages of the disease or when the lesion scope is limited, this is not always the case. In this study, two patients, one with lymphoma and the other with systemic lupus erythematosus, had extensive or multiple lesions; however, their serum CrAg test results were negative. Notably, BALF CrAg was also negative in the patient with lymphoma. Nevertheless, both patients tested positive for CrAg in the LPF samples, indicating that LPF-based CrAg detection may offer superior sensitivity. Additionally, serum/LPF-CrAg testing and traditional culture methods exhibited high specificity. Although primary lung cancer was common in our control group, other relevant conditions (bacterial pneumonia, PT, and aspergillosis, etc.) were also included. The specificity of the serum and LPF CrAg levels remained high even after excluding primary lung cancer cases, suggesting that this did not artificially inflate the results. One patient with PT had a false-positive serum CrAg result, whereas no false-positive results were observed in the patients with other pulmonary conditions. False-positive LAT results have been reported in patients with tuberculosis (Wang et al., 2023), whereas false-positive LFA results are rarely reported (Yamashiro-Kanashiro et al., 2025).

Recently, advancements in metagenomic next-generation sequencing (mNGS) and targeted next-generation sequencing (tNGS) technologies have led to their increasing application in the diagnosis of cryptococcal infections (Lai et al., 2005; Guo et al., 2021; Qin et al., 2025). However, owing to inherent challenges such as the thick fungal cell wall, which hinders efficient lysis and results in suboptimal nucleic acid extraction, the sensitivity of mNGS for PC detection is highly variable, ranging from 44.1% to 80.0% (Guo et al., 2021; Boyd et al., 2022; Huang et al., 2024). Moreover, mNGS has not been shown to perform better than serum CrAg testing (Huang et al., 2024), and some studies have shown inferior performance (Da et al., 2025). Similarly, tNGS has not been shown to perform better than serum CrAg testing for the detection of *Cryptococcus* (Huang et al., 2025). Notably, our previous study demonstrated that combining serum CrAg testing with mNGS analysis of lung specimens could improve diagnostic sensitivity to 93.3% (Huang et al., 2024). However, the combined use of serum and LPF CrAg testing offers distinct advantages in terms of the overall sensitivity, cost-effectiveness, and TAT. Regardless of the laboratory method used, including CrAg, the test results should be interpreted in conjunction with clinical findings to prevent overdiagnosis and missed diagnosis. The antigen detection reagent used in this study was an FDA-approved IMMY assay with high diagnostic accuracy (Macrae et al., 2023). However, multiple CrAg detection kits are currently commercially available, and their diagnostic performance varies (Chang et al., 2024; Kwizera et al., 2024), with some having suboptimal sensitivity or specificity.

This study has several limitations. First, this was a single-center study with a relatively small sample size. Therefore, our findings may not be reliable. Formal power analysis was not performed prospectively because this was an exploratory diagnostic accuracy study. This study was not powered for formal statistical comparisons between tests. Therefore, the findings, especially those from subgroup analyses, should be considered preliminary and require confirmation in larger studies. Second, to obtain sufficient volumes of LPF for concurrent routine pathogen culture, *Mycobacterium tuberculosis* testing, and other diagnostic assays, all specimens were diluted to 5 mL with normal saline. This dilution might have reduced the antigen concentration and affected the positivity rate. Furthermore, an inadequate volume of undiluted LPF in some patients may cause false-negative LPF CrAg test results in PC. Nevertheless, the performance of LPF samples was better than that of serum samples. Optimizing the dilution protocol and specimen collection quality in subsequent studies could further improve the sensitivity. Third, although cryptococcal lesions are predominantly located in the peripheral lung regions, many patients in this study underwent both bronchoscopy and PLB. However, the diagnostic value of routine BALF testing for CrAg has not yet been evaluated. Incorporating CrAg testing of multiple specimen types, including BALF, may further enhance diagnostic yield and maximize clinical utility. Fourth, LFA is suitable as a point-of-care test but has limited ability to quantify antigen reactivity (Schub et al., 2021). Although semi-quantitative detection can be achieved through repeated dilution of the specimen, this approach entails high reagent consumption. Finally, pneumothorax occurred in 6% of the patients, and hemoptysis occurred in 4.8% of the patients who underwent PLB. However, the incidence of these complications was comparable with that observed in a similar study (Hu et al., 2022) and remained low, as evidenced by findings reported in the existing literature (Zlevor et al., 2021). Moreover, all complications were mild and manageable. Pneumothorax resolved with oxygen therapy or drainage, and hemoptysis stopped spontaneously or with basic treatment. Importantly, the diagnostic yield was substantial, with a sensitivity of 97.0% for PC using combined serum and LPF CrAg testing. The diagnostic gain of LPF CrAg was highest in patients with an intermediate pre-test probability, where a positive result could potentially avert more invasive procedures such as surgical biopsy. For patients with a low pre-test probability, clinical/imaging features and the risk level of complications were carefully evaluated before PLB, and the procedure was only performed when PC was a significant consideration. The high diagnostic accuracy justified the minimal procedural risks in our cohort.

## Conclusion

In conclusion, this study demonstrates that serum CrAg testing has superior performance to conventional LPF *Cryptococcus* culture and that CrAg testing of LPF samples further enhances diagnostic sensitivity. Moreover, the combined detection of CrAg in both serum and LPF specimens has high sensitivity and specificity, thereby significantly improving the diagnostic efficiency. Integrating CrAg detection with respiratory interventional techniques facilitates the early, timely, and accurate diagnosis of PC and has a favorable safety profile. Our findings support its potential clinical application, although further prospective multicenter validations are required.

## Data Availability

The original contributions presented in the study are included in the article/Supplementary material, further inquiries can be directed to the corresponding authors.

## References

[B1] Botts M. R. Hull C. M. (2010). Dueling in the lung: How *Cryptococcus spores* race the host for survival. *Curr. Opin. Microbiol.* 13 437–442. 10.1016/j.mib.2010.05.003 20570552 PMC2920366

[B2] Boyd K. Kouamou V. Hlupen I. A. Tangwena Z. Ndhlovu C. E. Makadzange A. T. (2022). Diagnostic accuracy of point of care cryptococcal antigen lateral flow assay in fingerprick whole blood and urine samples for the detection of asymptomatic cryptococcal disease in patients with advanced HIV disease. *Microbiol. Spectr.* 10:e0107522. 10.1128/spectrum.01075-22 35924841 PMC9430595

[B3] Cáceres D. H. Zuluaga A. Tabares ÁM. Chiller T. González Á Gómez B. L. (2017). Evaluation of a cryptococcal antigen lateral flow assay in serum and cerebrospinal fluid for rapid diagnosis of cryptococcosis in Colombia. *Rev. Inst. Med. Trop. Sao Paulo* 59:e76. 10.1590/S1678-9946201759076 29267584 PMC5738761

[B4] Chang C. C. Harrison T. S. Bicanic T. A. Chayakulkeeree M. Sorrell T. C. Warris A. (2024). Global guideline for the diagnosis and management of cryptococcosis: An initiative of the ECMM and ISHAM in cooperation with the ASM. *Lancet Infect. Dis.* 24 e495–e512. 10.1016/S1473-3099(23)00731-4 38346436 PMC11526416

[B5] Chen L. She D. Liang Z. Liang L. Chen R. Ye F. (2021). A rospective multi-center clinical investigation of HIV-negative pulmonary cryptococcosis in China. *Zhonghua Jie He He Hu Xi Za Zhi* 44 14–27. 10.3760/cma.j.cn112147-20200122-00034 33412620

[B6] Chen M. Chen S. Wang M. Wang H. Zeng S. Zhuang L. (2025). A multicenter prospective clinical cohort study of pulmonary cryptococcosis in adult non-HIV-infected patients in a southeastern province of China. *Respir. Res.* 26:216. 10.1186/s12931-025-03283-w 40514686 PMC12164135

[B7] Chinese Thoracic Society of Chinese Medical Association (2017). Chinese expert consensus on pathogen detection in bronchoalveolar lavage fluid for pulmonary infectious diseases (2017 Edition). *Zhonghua Jie He He Hu Xi Za Zhi* 40 578–583. 10.3760/cma.j.issn.1001-0939.2017.08.007

[B8] Coussement J. Heath C. H. Roberts M. B. Lane R. J. Spelman T. Smibert O. C. (2023). Current epidemiology and clinical features of *Cryptococcus* infection in patients without human immunodeficiency virus: A multicenter study in 46 hospitals in Australia and New Zealand. *Clin. Infect. Dis.* 77 976–986. 10.1093/cid/ciad321 37235212

[B9] Da H. Meng T. Xu Y. (2025). Application of targeted next-generation sequencing for detecting respiratory pathogens in the sputum of patients with pulmonary infections. *Infect. Genet. Evol.* 128:105722. 10.1016/j.meegid.2025.105722 39909152

[B10] Donnelly J. P. Chen S. C. Kauffman C. A. Steinbach W. J. Baddley J. W. Verweij P. E. (2020). Revision and update of the consensus definitions of invasive fungal disease from the European organization for research and treatment of cancer and the mycoses study group education and research consortium. *Clin. Infect. Dis.* 71 1367–1376. 10.1093/cid/ciz1008 31802125 PMC7486838

[B11] Guo Y. Li H. Chen H. Li Z. Ding W. Wang J. (2021). Metagenomic nextgeneration sequencing to identify pathogens and cancer in lung biopsy tissue. *EBioMedicine* 73:103639. 10.1016/j.ebiom.2021.103639 34700283 PMC8554462

[B12] Hu Q. Li X. Zhou X. Zhao C. Zheng C. Xu L. (2022). Clinical utility of cryptococcal antigen detection in transthoracic needle aspirate by lateral flow assay for diagnosing non-HIV pulmonary cryptococcosis: A multicenter retrospective study. *Medicine* 101:e30572. 10.1097/MD.0000000000030572 36123876 PMC9478314

[B13] Huang J. Weng H. Ye L. Jiang M. Chen L. Li Y. (2024). Bronchoalveolar lavage fluid and lung biopsy tissue metagenomic next-generation sequencing in the diagnosis of pulmonary cryptococcosis. *Front. Cell. Infect. Microbiol.* 14:1446814. 10.3389/fcimb.2024.1446814 39534702 PMC11554620

[B14] Huang J. Ye L. Weng H. Jiang M. Lin Y. Li H. (2025). Targeted next-generation sequencing using bronchoalveolar lavage fluid samples for diagnosing pulmonary infections: A single-center retrospective study. *Front. Microbiol.* 16:1671819. 10.3389/fmicb.2025.1671819 41158766 PMC12554695

[B15] Kwizera R. Kiiza T. K. Akampurira A. Kimuda S. Mugabi T. Meya D. B. (2024). Evolution of laboratory diagnostics for cryptococcosis and missing links to optimize diagnosis and outcomes in resource-constrained settings. *Open Forum Infect. Dis.* 11:ofae487. 10.1093/ofid/ofae487 39282635 PMC11398909

[B16] Lai G. Zhang Y. Lin Q. Liu D. (2005). Domestic retrospective analysis of pulmonary cryptococcosis in the recent 22 years. *Chin. J. Pract. Int. Med.* 25 176–178. 10.3969/j.issn.1005-2194.2005.02.032

[B17] Lan C. Weng H. Li H. Chen L. Lin Q. Liu J. (2016). Retrospective analysis of 117 cases of pulmonary cryptococcosis. *Zhonghua Jie He He Hu Xi Za Zhi* 39 862–865. 10.3760/cma.j.issn.1001-0939.2016.11.008 27852362

[B18] Liaw Y. S. Yang P. C. Yu C. J. Chang D. B. Wang H. J. Lee L. N. (1995). Direct determination of cryptococcal antigen in transthoracic needle aspirate for diagnosis of pulmonary cryptococcosis. *J. Clin. Microbiol.* 33 1588–1591. 10.1128/jcm.33.6.1588-1591.1995 7650192 PMC228221

[B19] Liu Y. Kang M. Wu S. Wu L. He L. Xiao Y. (2022). Evaluation of a *Cryptococcus* capsular polysaccharide detection FungiXpert LFA (lateral flow assay) for the rapid diagnosis of cryptococcosis. *Med. Mycol.* 60:myac020. 10.1093/mmy/myac020 35362524

[B20] Liu Y. She D. Sun T. Tong Z. He B. Xiao Y. (2011). A multicentre retrospective study of pulmonary mycosis clinically proven from 1998 to 2007. *Zhonghua Jie He He Hu Xi Za Zhi* 34 86–90. 10.3760/cma.j.issn.1001-0939.2011.12.004 21426723

[B21] Liu Y. Wu W. Xiao Y. Zou H. Hao S. Jiang Y. (2024). Application of metagenomic next-generation sequencing and targeted metagenomic nextgeneration sequencing in diagnosing pulmonary infections in immunocompetent and immunocompromised patients. *Front. Cell. Infect. Microbiol.* 14:1439472. 10.3389/fcimb.2024.1439472 39165919 PMC11333343

[B22] Macrae C. Ellis J. Keddie S. H. Falconer J. Bradley J. Keogh R. (2023). Diagnostic performance of the IMMY cryptococcal antigen lateral flow assay on serum and cerebrospinal fluid for diagnosis of cryptococcosis in HIV-negative patients: A systematic review. *BMC Infect. Dis.* 23:209. 10.1186/s12879-023-08135-w 37024842 PMC10080957

[B23] May R. C. Stone N. R. Wiesner D. L. Bicanic T. Nielsen K. (2016). *Cryptococcus*: From environmental saprophyte to global pathogen. *Nat. Rev. Microbiol.* 14 106–117. 10.1038/nrmicro.2015.6 26685750 PMC5019959

[B24] Oshima K. Takazono T. Saijo T. Tashiro M. Kurihara S. Yamamoto K. (2018). Examination of cryptococcal glucuronoxylomannan antigen in bronchoalveolar lavage fluid for diagnosing pulmonary cryptococcosis in HIV-negative patients. *Med. Mycol.* 56 88–94. 10.1093/mmy/myx010 28419364

[B25] Pappas P. G. Perfect J. R. Cloud G. A. Larsen R. A. Pankey G. A. Lancaster D. J. (2001). Cryptococcosis in human immunodeficiency virus-negative patients in the era of effective azole therapy. *Clin. Infect. Dis.* 33 690–699. 10.1086/322597 11477526

[B26] Park B. J. Wannemuehler K. A. Marston B. J. Govender N. Pappas P. G. Chiller T. M. (2009). Estimation of the current global burden of cryptococcal meningitis among persons living with HIV/AIDS. *AIDS* 23 525–530. 10.1097/QAD.0b013e328322ffac 19182676

[B27] Perfect J. R. Bicanic T. (2015). Cryptococcosis diagnosis and treatment: What do we know now. *Fungal Genet. Biol.* 78 49–54. 10.1016/j.fgb.2014.10.003 25312862 PMC4395512

[B28] Qin W. Guo Y. Liang Z. Wei H. Huang H. Zhou L. (2025). *Cryptococcus* neoformans tibial osteomyelitis in an immunocompetent host: A case diagnosed by tNGS. *BMC Infect. Dis.* 25:1155. 10.1186/s12879-025-11451-y 41013340 PMC12465621

[B29] Schub T. Forster J. Suerbaum S. Wagener J. Dichtl K. (2021). Comparison of a lateral flow assay and a latex agglutination test for the diagnosis of *Cryptococcus* neoformans infection. *Curr. Microbiol.* 78 3989–3995. 10.1007/s00284-021-02664-w 34581848 PMC8486725

[B30] Setianingrum F. Rautemaa-Richardson R. Denning D. W. (2019). Pulmonary cryptococcosis: A review of pathobiology and clinical aspects. *Med. Mycol.* 57 133–150. 10.1093/mmy/myy086 30329097

[B31] Shi J. Chen J. Hu L. Ma A. Hu H. Wang C. (2023). Retrospective analysis of pulmonary cryptococcosis and extrapulmonary cryptococcosis in a chinese tertiary hospital. *BMC Pulm. Med.* 23:277. 10.1186/s12890-023-02578-2 37501136 PMC10375642

[B32] Shi Y. (2009). Diagnosis and management of pulmonary cryptococcosis. *Zhonghua Jie He He Hu Xi Za Zhi* 32 551–554. 10.3760/cma.j.issn.1001-0939.2009.07.025

[B33] Shirley R. M. Baddley J. W. (2009). Cryptococcal lung disease. *Curr. Opin. Pulm. Med.* 15 254–260. 10.1097/MCP.0b013e328329268d 19352182

[B34] Su Y. Miao Q. Li N. Hu B. Pan J. (2022). Diagnostic accuracy of metagenomic next-generation sequencing for cryptococcosis in immunocompetent and immunocompromised patients. *Front. Cell. Infect. Microbiol.* 12:997256. 10.3389/fcimb.2022.997256 36339336 PMC9630913

[B35] Wang H. Yan S. Liu Y. Li Y. Cui G. Ma X. (2022). Metagenomic next-generation sequencing assists in the diagnosis of Cryptococcus pneumonia: Case series and literature review. *Front. Public Health* 10:971511. 10.3389/fpubh.2022.971511 36408040 PMC9672815

[B36] Wang Y. Zhang Y. Du J. Yao W. Qin Q. Wang H. (2023). Analysis of interference factors in various detection methods for cryptococcal capsular polysaccharide antigen. *Chin. J. Clin. Lab. Sci.* 41 68–71. 10.13602/j.cnki.jcls.2023

[B37] Weng H. (2010). Expert consensus on diagnosis and treatment of cryptococcal infection. *Chin. J. Mycol.* 5:86. 10.3969/j.issn.1673-3827.2010.02.001

[B38] World Health Organization (2022). *WHO fungal priority pathogens list to guide research, development and public health action.* Geneva: WHO.

[B39] Xie H. Zhang Y. Wu Z. Li S. Zhou S. Liu L. (2017). Diagnostic value of lateral flow immunoassay and latex agglutination test in AIDS patients with infection. *Fujian Med. J.* 9 96–99. 10.20148/j.fmj.2017.03.032

[B40] Yamashiro-Kanashiro E. H. Kanunfre K. A. Mimicos E. V. de Freitas V. L. T. Rocha M. C. Shimoda Nakanishi ÉY. (2025). Reactivity of cryptococcal lateral flow assay in aspergillosis, histoplasmosis, paracoccidioidomycosis, candidiasis, trichosporonosis, bacterial, and viral infections. *Med. Mycol.* 63:myaf068. 10.1093/mmy/myaf068 40699015

[B41] Yan Q. Sun Z. Gao Y. Xiao T. Lin H. Ji M. (2021). Diagnostic value of the combinations of bronchoalveolar lavage fluid pathogen detection and cryptococcal antigen test in pulmonary cryptococcosis. *Zhonghua Jie He He Hu Xi Za Zhi* 44 711–716. 10.3760/cma.j.cn112147-20201123-01113 34645137

[B42] Zeng H. Zhang X. Cai X. Yang D. Lin L. Chen M. (2021). Diagnostic value of bronchoalveolar lavage fluid cryptococcal antigen-lateral flow immunochromatographic assay for pulmonary cryptococcosis in non-HIV patients. *Diagn. Microbiol. Infect. Dis.* 99:115276. 10.1016/j.diagmicrobio.2020.11527 33341492

[B43] Zheng Y. Qiu X. Wang T. Zhang J. (2021). The diagnostic value of metagenomic next-generation sequencing in lower respiratory tract infection. *Front. Cell Infect. Microbiol.* 11:694756. 10.3389/fcimb.2021.694756 34568089 PMC8458627

[B44] Zhou J. Yu Y. Zhejiang Medical Association Society of Respiratory Diseases (2017). Expert consensus on diagnosis and treatment of pulmonary cryptococcosis. *Chin. J. Clin. Infect. Dis.* 10 321–326. 10.3760/cma.j.issn.1674-2397.2017.05.001

[B45] Zlevor A. M. Mauch S. C. Knott E. A. Pickhardt P. J. Mankowski Gettle L. Mao L. (2021). Percutaneous lung biopsy with pleural and parenchymal blood patching: Results and complications from 1,112 core biopsies. *J. Vasc. Interv. Radiol.* 32 1319–1327. 10.1016/j.jvir.2021.06.022 34229043

