# Supplementary material for: Diagnostic value of combined detection of cryptococcal antigen in serum and lung puncture fluid using lateral flow assay for diagnosing pulmonary cryptococcosis: a single-center prospective study
Source: Front Microbiol. 2026 Jan 16;16:1747123. doi: 10.3389/fmicb.2025.1747123 (PMC12855499; doi:10.3389/fmicb.2025.1747123)
Supplement: Supplementary file 1 [file Supplementary_file_1.docx]

Supplementary Table 1. Comparison of serum cryptococcal antigen (CrAg) results among patients with pulmonary cryptococcosis according to immunological and radiological characteristics

| Immunological and radiological characteristics | Positive for serum CrAg (N = 25) n (%) | Negative for serum CrAg (N = 8) n (%) | *P* |
| --- | --- | --- | --- |
| Immune conditions |  |  |  |
| Underlying disease | 16 (64) | 6 (75) | 0.687 |
| Immunosuppressive disease | 12 (48) | 5 (62.5) | 0.688 |
| Symptoms |  |  |  |
| Asymptomatic | 17 (68) | 6 (75) | >0.999 |
| Chest CT results |  |  |  |
| Distribution of pulmonary lesions |  |  |  |
| Isolated lesion | 6 (24) | 4 (50) | 0.205 |
| Multiple lesions in a single lobe | 15 (68) | 7 (87.5) | 0.218 |
| Multiple lesions in one lung | 17 (67) | 7 (87.5) | 0.394 |
| Imaging type |  |  |  |
| Nodule and/or mass | 19 (76) | 5 (62.5) | 0.651 |
| Isolated nodule | 3 (16) | 3 (60) | 0.078 |
| Consolidation | 1 (4) | 1 (12.5) | 0.432 |
| Mixed lesions | 5 (20) | 2 (25) | >0.999 |
| Signs |  |  |  |
| Halo | 10 (40) | 2 (25) | 0.378 |
| Vacuole or cavity | 6 (24) | 3 (37.5) | 0.651 |
| Air bronchogram | 7 (28) | 1 (12.5) | 0.643 |
| Lobulation | 6 (24) | 2 (25) | >0.999 |
| Spiculation | 3 (12) | 1 (12.5) | >0.999 |
| Pleural indentation | 2 (8) | 0 (0) | >0.999 |
| Vessel convergence | 2 (8) | 0 (0) | >0.999 |
| Pleural effusion | 2 (8) | 0 (0) | >0.999 |

Abbreviations: CrAg, *Cryptococcus* capsular polysaccharide antigen; CT, computed tomography; PC, pulmonary cryptococcosis

Data are presented as n (%).

Significance was determined using the chi-squared test or or Fisher’s exact test.
