# Supplementary figures and images for: Diagnostic value of combined detection of cryptococcal antigen in serum and lung puncture fluid using lateral flow assay for diagnosing pulmonary cryptococcosis: a single-center prospective study
Source: Front Microbiol. 2026 Jan 16;16:1747123. doi: 10.3389/fmicb.2025.1747123 (PMC12855499; doi:10.3389/fmicb.2025.1747123)

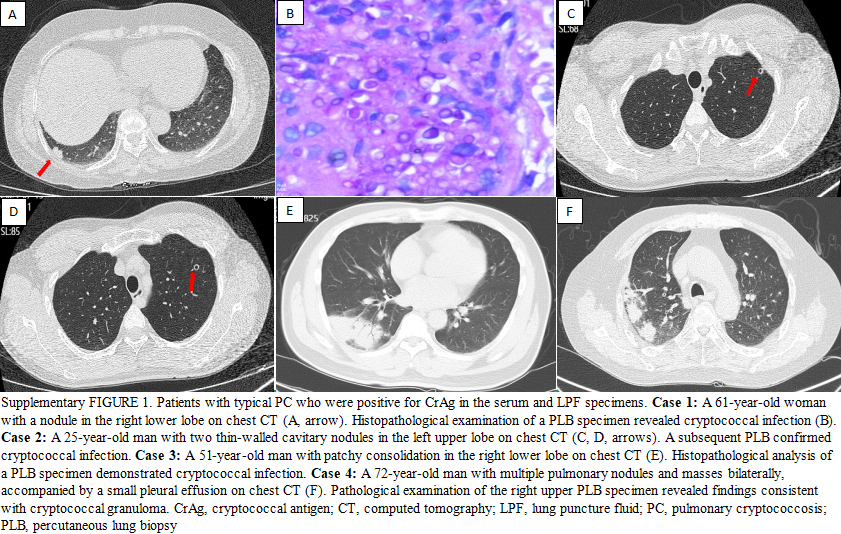

Supplement: Supplementary file 2 [file Image_1.tif]

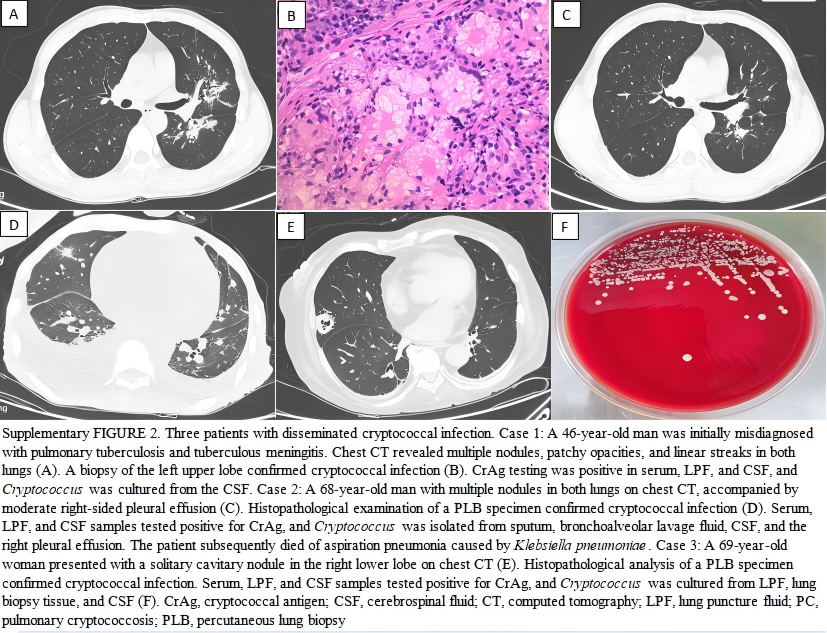

Supplement: Supplementary file 3 [file Image_2.tif]
